# Attitudes and Engagement of Pregnant and Postnatal Women With a Web-Based Emotional Health Tool (Mummatters): Cross-sectional Study

**DOI:** 10.2196/18517

**Published:** 2021-03-26

**Authors:** Nicole Reilly, Marie-Paule Austin

**Affiliations:** 1 Centre for Health Service Development Australian Health Services Research Institute University of Wollongong Wollongong Australia; 2 Perinatal & Women's Mental Health Unit St John of God Burwood Hospital & School of Psychiatry University of New South Wales Burwood Australia; 3 Royal Hospital for Women Randwick Australia

**Keywords:** pregnancy, postpartum, self-assessment, depression, risk

## Abstract

**Background:**

*Mummatters* is a web-based health tool that allows women to self-assess the symptoms of depression and the presence of psychosocial risk factors throughout pregnancy and the postnatal period. It aims to increase women’s awareness of their own symptoms or risk factors and their knowledge of the available support options, to encourage engagement with these support options (as appropriate), and to facilitate communication about emotional health issues between women and their health care providers.

**Objective:**

The aim of this study is to report the uptake of *mummatters*; the sociodemographic and psychosocial risk profiles of a subsample of users; and the acceptability, credibility, perceived effect, and motivational appeal of the tool. The help-seeking behaviors of the subsample of users and barriers to help seeking were also examined.

**Methods:**

*Mummatters* was launched in November 2016. Women who completed the *mummatters* baseline assessment were invited to complete a web-based follow-up survey 1 month later.

**Results:**

A total of 2817 women downloaded and used *mummatters* between November 13, 2016, and May 22, 2018, and 140 women participated in the follow-up study. Approximately half of these women (51%; 72/140) were *Whooley positive* (possible depression), and 43% (60/140) had an elevated psychosocial risk score on the Antenatal Risk Questionnaire. *Mummatters* was rated favorably by pregnant and postnatal women in terms of its acceptability (94%-99%), credibility (93%-97%), appeal (78%-91%), and potential to affect a range of health behaviors specific to supporting emotional wellness during the perinatal period (78%-93%). *Whooley-positive* women were more likely to speak with their families than with a health care provider about their emotional health. Normalizing symptoms and stigma were key barriers to seeking help.

**Conclusions:**

Although *mummatters* was rated positively by consumers, only 53% (19/36) to 61% (22/36) of women with possible depression reported speaking to their health care providers about their emotional health. There was a trend for more prominent barriers to seeking help among postnatal women than among pregnant women. Future studies that investigate whether social barriers to seeking help are greater once a woman has an infant are warranted. Such barriers potentially place these women at greater risk of remaining untreated, as the demands on them are greater.

## Introduction

### Background

Overall, 1 in 7 women experience some form of mental health morbidity during pregnancy and the first postnatal year (the perinatal period) [1,2]. When left untreated, perinatal depression and anxiety may persist for years after birth and can affect not only the woman’s capacity to parent but also the emotional well-being of the infant and other family members [3,4]. The importance of early detection and intervention in this susceptible population has been widely acknowledged [5-9].

The health service systems in place for routine maternity care in Australia have provided a unique opportunity to introduce perinatal mental health promotion, prevention, and early intervention programs. These programs and related clinical guidelines aim for the early identification of possible or probable illness, or risk of illness, and then to monitor or intervene as appropriate, with a view to improve maternal mental health outcomes [6,10]. However, there are disparities in access to these programs, with women who give birth in the private maternity sector, for example, being less likely to be assessed across various domains of psychosocial health during pregnancy or the postpartum period [11-13].

In response to this inequity of access, Bupa Australia collaborated with perinatal mental health and consumer teams to develop a web-based tool, *mummatters* [14], which allows women to self-assess for the symptoms of depression and the presence of psychosocial risk factors throughout the perinatal period. *mummatters* aims to increase women’s awareness of their own symptoms or risk factors and their knowledge of the available support options, encourage engagement with these support options (as appropriate), and facilitate communication about emotional health issues between women and their health care providers.

### Objective

The aim of this study was to report on the uptake of *mummatters*; the sociodemographic and psychosocial risk profiles of a subsample of users; and the acceptability, credibility, perceived effect, and motivational appeal of the tool to pregnant and postnatal women. The help-seeking behaviors of women who used *mummatters* and barriers to help seeking were also examined.

## Methods

### Mummatters Overview

*Mummatters* is a web-based tool that is available free of charge via the Bupa website [14]. Its web-based design allows access from a range of computing and mobile devices. Users can bookmark the website or save *mummatters* to the home page of their devices, where it appears as an icon. Internet access is required to use the features of the tool.

After downloading *mummatters*, women are invited to answer a small number of demographic questions (including current gestation or infant age and maternity care sector), followed by a baseline assessment comprising the *Whooley* questions [15,16] and the Antenatal Risk Questionnaire (ANRQ; and its postnatal equivalent) [17].

The *Whooley* depression case-finding questions are recommended for use in the perinatal period by the National Institute for Health and Care Excellence [8]. The 2-item questionnaire has been shown to have a high sensitivity (0.95; 95% CI 0.88-0.97) and modest specificity (0.65; 95% CI 0.56-0.74) [18]. The *Whooley* questions are as follows: (1) “During the past month, have you often been bothered by feeling down, depressed or hopeless?” and (2) “During the past month, have you often been bothered by having little interest or pleasure in doing things?” [15]. These depression-related questions are followed by a third question that is asked to women who responded “yes” to either of the 2 above-mentioned questions (“Is this something you feel you need or want help with?”) [16]. Women were considered to be *Whooley positive* (possible depressive episode) if they answered “yes” to either/both questions 1 or 2 [8].

The ANRQ is a validated self-report measure that was developed by a panel of experts, based on evidence relating to salient risk factors associated with perinatal mental health disorders, particularly depression and anxiety, and on the face and construct validity of these factors. Its capacity to identify women at increased risk for these conditions has been demonstrated [17]. Although initially developed for the antenatal period, the ANRQ has been used during the postnatal period for research and clinical practice [19,20]. Australia’s current clinical practice guidelines for mental health care in the perinatal period recommend the use of the ANRQ for the assessment of psychosocial risk [6].

A key feature of *mummatters* is its computer-based decision aid that combines responses to the Whooley questions and ANRQ to generate tailored follow-up messages and provide help-seeking information, as appropriate. For example, women who are *Whooley positive* automatically receive a message encouraging them to make an appointment to discuss their emotional health with a trusted health care professional. The tool also allows women to give permission for a letter addressed to their health care provider to be generated, which includes a summary of their results as well as full copies of their completed measures. Women are also given ready access to additional information and links to resources that aim to support them in actively looking after their emotional well-being. After the completion of the initial baseline assessment, *mummatters* sends monthly prompts for women (irrespective of their baseline scores) to complete follow-up assessments to monitor their emotional health and well-being. Women can also create an individualized wellness action plan and can opt to receive inspirational messages sent monthly via SMS or email.

### Data Collection and Research Participants

There were 2 primary sources of data for this evaluation. The first was *mummatters* use data that women consented to being used for research purposes via a within-tool agreement. These use data included a unique identification number as well as deidentified demographic and clinical information provided during the initial pregnancy or postnatal assessment.

The second data source was a research-specific data set that required additional consent. All women who used *mummatters* at least once during pregnancy or postnatally and who indicated their willingness to be contacted about the study were emailed and invited to participate. To be eligible, women were also required to be currently living in Australia, have access to the internet, and be able to complete the measures in English. Eligible women who agreed to participate gave informed consent and completed the additional study measures via the web-based *Key Survey* (TM) platform. A reminder email was sent by the research team to women who did not complete the study measures within 1 week, with 2 further reminders sent at weekly intervals thereafter (up to a maximum of 3 reminders).

Participants completed questions relating to the acceptability, credibility, likeability, perceived effect, and motivational appeal of the tool. Participants were also asked about help-seeking behaviors in the previous month and barriers to help seeking. Where possible, these questions were replicated or modified from previous studies for methodological consistency [21,22]. These data were linked to the use data of the participants via their unique identification numbers.

### Ethical Approval

The study was approved by the Human Research Ethics Committee (HREC) of St John of God Health Care (HREC reference number: 735).

## Results

### Sociodemographic and Psychosocial Profile of Mummatters Evaluation Participants

A total of 2817 women downloaded and completed the *mummatters* baseline measures between November 13, 2016, and May 22, 2018. Of these, 26.80% (755/2817) indicated their willingness to be contacted about the study and were emailed an information sheet, consent form, and link to the study measures approximately 4 weeks after indicating their expression of interest. Of these, 33.8% (255/755) women agreed to participate in the study; among these, 91 women dropped out of the survey immediately after indicating their consent (ie, before completing any of the research questions). Of the remaining 164 women, 140 had sufficient research data and were subsequently included in the analyses (ie, 5% (140/2817) of all *mummatters* users and 18.5% (140/755) of all women emailed about the research). The demographic profiles of the 140 women who participated are presented in Table 1.

There were no significant differences between pregnant or postnatal *mummatters* users who were and were not included in the study in terms of *Whooley-positive* status (antenatal: *χ*^2^(2)=1.0; *P*=.60 and postnatal: *χ*^2^(2)=2.5; *P*=.29), ANRQ total score (antenatal: *t_1331_*=−1.32; *P*=.19 and postnatal: *t_1369_*=−0.05; *P*=.96), private maternity sector (antenatal: *χ*^2^(2)=1.1; *P*=.78 and postnatal: *χ*^2^(1)=0.7; *P*=.68), gestation (*t_1377_*=0.34; *P*=.73), or infant age (*t_1433_*=1.38; *P*=.17) at baseline assessment.

The results of the *mummatters* baseline psychosocial assessment (Whooley questions and psychosocial risk questionnaire) for participants are summarized in Table 2. During pregnancy, 49% (n=36/73) of participants were *Whooley positive*; one or both of the Whooley questions were endorsed by 15% (11/73) and 34% (25/73) of participating women, respectively. In the postnatal period, 36 of 67 (54%) participants were *Whooley positive*, with 31% (21/67) of women endorsing one question and 22% (15/67) endorsing both questions. Across the perinatal period, women who endorsed both Whooley questions were significantly more likely to answer “yes” to the third Whooley *help* question than women who endorsed one question only (antenatal: *χ*^2^(1)=5.7; *P*=.02 and postnatal: *χ*^2^(1)=5.2; *P*=.02).

**Table 1:** Sociodemographic characteristics of mummatters evaluation participants.

Characteristic			Antenatal period (baseline; n=73)		Postnatal period (baseline; n=67)
**Gestation or infant age at first** *mummatters* **baseline assessment (weeks)**					
	Mean (SD)	20.96 (11.19)		15.28 (24.11)	
	Range	4-40		1-178	
**Maternal age (years)**					
	Mean (SD)	32.97 (4.60)		32.70 (4.20)	
	Range	24-43		25-45	
Partnered^a^, n (%)			68 (96)		65 (99)
First child^b^, n (%)			42 (62)		28 (44)
Australian born^b^, n (%)			60 (88)		49 (78)
**Maternity sector^c^, n (%)**					
	Public	32 (44)		30 (46)	
	Private	37 (51)		36 (55)	
	Undecided	4 (6)		N/A^d^	
**Residential area^a^, n (%)**					
	Metropolitan	51 (72)		43 (65)	
	Regional	14 (20)		20 (30)	
	Rural	6 (9)		3 (5)	
**Highest educational level^a^, n (%)**					
	Highschool	6 (9)		4 (6)	
	TAFE^e^ or diploma	20 (28)		15 (23)	
	Bachelor’s degree	30 (42)		28 (42)	
	Postgraduate degree	15 (21)		19 (29)	
**Current employment status^a^, n (%)**					
	On maternity leave	19 (27)		45 (68)	
	Full time	33 (47)		4 (6)	
	Part time	12 (17)		6 (9)	
	Unemployed	7 (10)		11 (17)	
**Income management^a,f^, n (%)**					
	Difficult	17 (24)		26 (39)	
	Not difficult	54 (76)		40 (61)	

^a^Excludes 2 antenatal women and 1 postnatal woman with missing data.

^b^Excludes 5 antenatal women and 4 postnatal women with missing data.

^c^Excludes 1 postnatal woman with missing data.

^d^N/A: not applicable.

^e^TAFE: Technical and Further Education.

^f^Responses to the question “How do you manage on the income you have available” were divided into difficult (“It is impossible,” “It is difficult all of the time,” and “It is difficult some of the time”) or not difficult (“It is too bad” and “It is easy”) responses. Columns may not total 100% due to rounding.

**Table 2:** Psychosocial profile of mummatters evaluation participants.

Characteristic		Antenatal period (baseline; n=73)	Postnatal period (baseline; n=67)
**Psychosocial risk questionnaire (ANRQ^a^) total score**			
	Mean (SD)	23.11(10.64)	23.09 (10.00)
	Range	6-53	7-49
Whooley positive^b^, n (%)		36 (49)	36 (54)
ANRQ score≥23, n (%)		30 (41)	30 (45)
**ANRQ item, n (%)**			
	High trait anxiety (being a worrier)^c^	20 (27)	22 (33)
	Perfectionistic traits^c^	19 (26)	14 (21)
	Past sexual or physical abuse	17 (23)	9 (13)
	Significant past mental health issues^d^	31 (22)	18 (27)
	Major stressors in the last 12 months^e^	16 (22)	18 (27)
	Emotional abuse in childhood	14 (19)	11 (16)
	Growing up with emotionally unsupportive mother^c^	13 (18)	18 (27)
	Lack of support with the baby^c^	7 (10)	12 (18)
	Emotionally unsupportive partner^c^ (or no partner)	6 (8)	4 (6)

^a^ANRQ: Antenatal Risk Questionnaire.

^b^Endorsed one or both of the two Whooley questions.

^c^Antenatal Risk Questionnaire; scaled items were dichotomized into low scoring (1-3) or high scoring (4 or more), consistent with the methodology used in previous research [23,24].

^d^Item considered endorsed if participants responded “yes” to depression or other past mental health problems for which professional help was sought or which significantly interfered with work and relationships (score of 4 or more).

^e^Item considered endorsed if participants responded “yes” to experiencing a major stressor in the previous 12 months which caused a significant degree of distress (score of 4 or more).

Overall, 33% (24/73) of women who completed the antenatal baseline assessment did not endorse any significant risk factors on the ANRQ, 26% (19/73) endorsed 1 significant risk factor, 15% (11/73) endorsed 2 significant risk factors, and 26% (19/73) had 3 or more significant risk factors. Postnatally, 19% (13/67), 24% (16/67), 31% (21/67), and 25% (17/67) of women endorsed none, 1, 2, and 3 or more risk factors on the ANRQ, respectively. A total of 41% (30/73) of antenatal participants and 45% (30/67) postnatal participants scored 23 or more, which is the cutoff score for the ANRQ. Women who score above the cutoff are considered to be experiencing a significant accumulation of risk factors that are associated with an increased risk of developing a clinical depression or anxiety disorder [17].

### Participant Experience and Feedback

The mean time taken by women to complete the *mummatters* baseline questions was 4 minutes (antenatal women: mean 4.16 minutes, SD 11.27 minutes; postnatal women: mean 3.88 minutes, SD 8.48 minutes). Most pregnant and postpartum women rated *mummatters* favorably on a range of feedback parameters (Table 3). Of note, most pregnant and postnatal users regarded *mummatters* as acceptable (94.0%-98.6%), credible (93.2%-97.3%), appealing (78.1%-91.0%), and potentially helpful in affecting a range of health behaviors specific to supporting emotional wellness during the perinatal period (78.1%-92.5%).

**Table 3:** Participant agreement with feedback statements relating to the use of mummatters.

Feedback statement		Antenatal period (n=73), n (%)^a^	Postnatal period (n=67), n (%)^a^
**Acceptability**			
	I felt comfortable completing questions about my emotional health and well-being using *mummatters*	72 (99)	63 (94)
**Credibility**			
	The information I got from *mummatters* can be trusted	71 (97)	64 (96)
	The information I got from *mummatters* was useful	68 (93)	64 (96)
**Perceived effect**			
	The information in *mummatters* helped me better understand the importance of having good emotional health in the transition to motherhood	65 (89)	62 (93)
	*mummatters* helped me learn about the symptoms of depression	57 (78)	61 (91)
	*mummatters* helped me learn about some common risk factors for depression and anxiety during pregnancy and in the year after birth	63 (86)	61 (91)
	*mummatters* will help me pay closer attention to my emotional health and well-being	58 (80)	54 (81)
	*mummatters* would help me feel more comfortable in seeking support for emotional health issues during pregnancy and in the year after birth, if I needed it	62 (85)	55 (83)
	*mummatters* would help reduce the stigma of seeking help for emotional health issues during pregnancy and in the year after birth, if I needed it	65 (89)	56 (84)
	*mummatters* increased my awareness of additional resources for emotional well-being during pregnancy and in the year after birth	63 (86)	59 (88)
	*mummatters* provides practical solutions to managing emotional health issues during pregnancy and in the year after birth	60 (82)	60 (90)
	The report that I can download in *mummatters* would help me talk to my health care provider about my emotional well-being, if I needed to	63 (86)	58 (87)
	The information provided in *mummatters* could help me manage my emotional well-being in the future	63 (86)	61 (91)
**Motivational appeal**			
	I would use *mummatters* again	66 (90)	61 (91)
	I would tell friends to use *mummatters*	57 (78)	57 (85)
**Likeability**			
	It was easy to find the information I wanted in *mummatters*	66 (90)	59 (88)
	The information I got from *mummatters* was relevant to me	66 (90)	62 (93)
	Overall, the features of *mummatters* met my expectations	58 (80)	54 (81)

^a^Numbers and percentages indicate those who agreed or strongly agreed with each statement.

### Help-Seeking Behaviors and Barriers to Help Seeking

The help-seeking behaviors of *Whooley-positive* women (ie, women endorsing one or both of the Whooley depression questions) in the month before completing the study survey as well as the barriers to help seeking are presented in Table 4. Overall, these women were the most likely to discuss their emotional health with their partners or family during both pregnancy (33/36, 92%) and the postnatal period (30/36, 83%). Other common sources of support were friends (56%-61%), health care providers (53%-61%), and books or print materials (56%-64%). Women were more likely to report using complementary therapies for their emotional health during pregnancy than after birth. Interestingly, only 23% (5/22) of *Whooley-positive* women who spoke with a health care provider during pregnancy took their *mummatters* report to the appointment with them, which decreased to 11% (2/19) in the postnatal period.

**Table 4:** Help-seeking behaviors and barriers to help seeking in the month before completing the study survey among antenatal and postnatal Whooley-positive women.

Help-seeking behaviors and barriers		Antenatal period (n=36), n (%)	Postnatal period (n=36), n (%)	*P* value^a^
**Help-seeking behaviors in the** **month before completing the study survey**				
	Spoke to health care professional^b^	22 (61)	19 (53)	.47
	Partner or family	33 (92)	30 (83)	.48
	Friends	22 (61)	20 (56)	.54
	Internet	13 (36)	15 (42)	.42
	Books or print materials	20 (56)	23 (64)	.53
	Lifestyle changes	9 (25)	15 (42)	.10
	Complementary therapies (including supplements)	16 (44)	7 (19)	.04
	Started or continued medication	7 (19)	3 (8)	.19
	Phone helpline	0 (0)	3 (8)	.24
	Day stay or residential parenting service	N/A^c^	3 (8)	N/A
	Hospital emergency department or admission	0 (0)	2 (6)	.49
**Barriers to help-seeking in the** **month before completing the study survey**				
	Did not think needed help	7 (19)	11 (30)	.31
	Normalizing symptoms	11 (31)	17 (46)	.18
	Not aware of services	6 (17)	6 (16)	.96
	Would feel like a failure	6 (17)	12 (32)	.12
	Fear of judgment	10 (28)	17 (46)	.11
	Worried about side effects of treatment	6 (17)	4 (11)	.52
	Could not afford it	6 (17)	3 (8)	.31
	Could not arrange childcare or transport	0 (0)	4 (11)	.12

^a^Chi-square test was used when n is >5, and Fisher exact test was used when n is <5.

^b^Includes midwife, child health nurse, general practitioner, obstetrician, counselor, psychologist, and psychiatrist.

^c^N/A: not applicable.

The most common barriers to seeking additional help or support reported by *Whooley-positive* women were personal or social in nature. For example, 46% (17/36) of postnatal participants normalized their symptoms or feared that they would be negatively judged if they asked for help, and up to one-third of women reported that they would feel like a failure. The proportion of women reporting these effects of stigma was greater in the postnatal period than during pregnancy, although these differences were not statistically significant.

## Discussion

### Principal Findings

This study sought to report on the uptake of *mummatters* and to provide insights into the experience, psychosocial profile, and help-seeking behaviors of women who engaged with this free, web-based self-assessment tool. Approximately 3000 women downloaded *mummatters* in 18 months. The results demonstrated that the tool was positively appraised by both pregnant and postnatal users, with high levels of reported acceptability, credibility, likeability, perceived effect, and motivational appeal. Approximately half of the women who used *mummatters* had chosen private maternity care for their current pregnancy or birth (48%; 1358/2817), suggesting that the tool was reaching a population that was known to be less likely to be offered depression screening and psychosocial assessment as a routine component of their antenatal and postnatal care.

Existing Australian research has reported elevated ANRQ scores among 14%-32% of women [23-25] in the general perinatal population, and although there is no Australian comparison data available for the Whooley questions, previous community-based studies have reported *Whooley-positive* rates of between 10% and 51% [26,27]. In comparison, up to 45% of women in this study scored above the recommended clinical cutoff score on the ANRQ and up to 54% were *Whooley positive*, suggesting that women who are at greater risk of poorer emotional health or parenting outcomes or who are experiencing current symptoms of depression are using *mummatters* and finding it highly acceptable. In keeping with recent research, this pattern of results may also reflect that an anonymous web-based assessment makes it easier for women to give an honest account of how they are feeling [28,29]. Although depression screening and psychosocial assessment are largely acceptable to most women and health providers [17,23,24,30], recent research has shown that women who are most likely to need mental health care during the perinatal period are also those least likely to be honest with their health care providers when responding to questions about their mental health [31].

It is well-established that fear and shame are significant factors in women’s decisions to seek or accept help for mental health issues during the perinatal period [32]. Approximately half of the women normalized their symptoms and were particularly concerned with how others would judge them if they admitted that they were struggling emotionally with motherhood. The barriers presented by these stigmatizing beliefs remain despite general population community surveys showing a high rate of disagreement with negative stereotypes about depression and motherhood, including disagreeing with the view that women with postnatal depression are unable to be good mothers [33].

Up to 47% of *Whooley-positive* women in this study did not speak to a health care professional about how they were feeling in the month after completing the *mummatters* baseline assessment, and only a few (10.5%-22.7%) of those that did took the downloadable *mummatters* report to their appointment. Although this is in line with previous Australian research that shows that up to 50% of women do not seek help for emotional health issues during the perinatal period despite being identified as in need of additional support [34,35], there is a clear need to understand why the report was being underutilized and how this can be made more useful, particularly given that women had already taken a first step in seeking a way to evaluate their symptoms. Seeking women’s consent to automatically send the reports from *mummatters* to a nominated health care provider is one possible response so that the onus to seek care does not solely lie on the women, whose symptoms may inherently make it difficult to seek support. However, this option assumes that women will have continuity of care, but this is not always possible in contexts where care is delivered across hospital maternity care systems and postnatal community–based primary care systems and between the public and private health care sectors. Gathering women’s views on how this feature can be improved or made more acceptable to users was beyond the scope of this study but is critical to inform future updates of the tool. Consistent with other Australian research [22,35,36], this study showed that family and friends are key support options for many women during pregnancy and the postnatal period. This again highlights the critical importance of targeting partners, family, and social networks in community awareness campaigns and early intervention programs for perinatal mental health. However, such campaigns must be complemented by support and treatment approaches that are well resourced, available, and enhance timely access to appropriate follow-up care. Interestingly, both pregnant and postnatal women in this study were more likely to report seeking support or information about their emotional health from books or print materials than the internet. This was despite women already being engaged with *mummatters* as a web-based tool and despite the increasing availability of locally developed internet-based resources and interventions [37]. The feasibility of partnering with service providers to directly link women to evidence-based web-based and telehealth treatment programs in future iterations of *mummatters* is currently being explored.

### Strengths and Limitations

This study has several limitations. The sample size was small; however, based on the limited comparison data available, participants were representative of all *mummatters* users. Although it was a self-selected sample, a greater than expected proportion of participants endorsed possible depression or a substantial number of psychosocial risk factors, predisposing them to developing a mental health episode. Thus, we were able to examine the concerns of more susceptible women more closely in terms of help-seeking behaviors and barriers.

### Conclusions

Previous research has reported on the engagement and real-world clinical utility of web-based approaches to self-administered screening for mental health conditions [38-41]; however, such approaches have not been adequately evaluated in perinatal populations. This study provided insight into the profile, experience, and help-seeking behaviors of women who used *mummatters*, a freely available web-based tool, and our results will help inform the review and further development of the tool. Although *mummatters* was rated positively by consumers, only 53% (19/36) to 61% (22/36) of women with possible depression reported speaking to their health care provider about this. This was more notable (though not statistically significant) among postnatal women than among pregnant women, suggesting that the barriers to help seeking are greater once a woman has an infant. Such barriers potentially place these women at greater risk of remaining untreated, as the demands on them are greater. This warrants further investigation in future studies.

Although consumer-driven risk assessments and symptom checklists are becoming more readily available for perinatal women, the need to keep training health care providers to engage women and ask the right questions to start the conversation around emotional well-being remains imperative [42]. Multiple but complementary approaches may be necessary given the well-documented findings, supported by our study, that many women do not seek formal assistance from their health care providers even when they are encouraged to do so. Future research should also focus on whether engagement with self-assessment tools of this type, including frequency and duration of engagement, is associated with longer-term improvements in mental health and health-related quality of life.

## Abbreviations

ANRQ: Antenatal Risk Questionnaire
HREC: Human Research Ethics Committee
